# The Multidimensional Assessment of Parenting Scale: Youth Report Form in Inpatient and Partial Hospital Settings

**DOI:** 10.1007/s10826-025-03131-x

**Published:** 2025-08-12

**Authors:** Christina M. Hogan, Emily Beckmann, Micaela Maron, Kelsey Sutton, April Highlander, Melissa Pielech, Jennifer C. Wolff, Thamara Davis, Justin Parent

**Affiliations:** 1 University of Massachusetts Amherst, Amherst, MA USA; 2 Warren Alpert Medical School at Brown University, Providence, RI USA; 3 Emma Pendleton Bradley Hospital, East Providence, RI USA; 4 University of Rhode Island, Kingston, RI USA

**Keywords:** Measurement, Parenting, Children, Adolescents, Inpatient

## Abstract

The Multidimensional Assessment of Parenting Scale (MAPS) was developed to assess a wide range of behaviors across positive and negative domains of parenting. This study aims to expand the utility of the MAPS by evaluating a youth-report version which provides an additional perspective on parenting practices. The study evaluated the youth-report form of the MAPS (MAPS-Y) in a large clinical population (*N* = 628) ranging from middle childhood (8–12) to adolescence (13–17) who were admitted to partial and inpatient psychiatric units. Youth and their caregivers completed the parent and youth versions of the MAPS questionnaire, and measures of child and adolescent psychopathology, emotion regulation, family context, and adversity. Analyses of factor structure, reliability, agreement, and validity were performed. The study also examined a short form of the MAPS-Y for reliability and validity. CFA and model fit indices indicated that all items loaded as expected onto subscales and with good fit. Analyses support strong reliability. The factor structure of the youth-report form was invariant across developmental stages, included both positive and negative domains, and demonstrated strong psychometric properties. The MAPS-Y short form demonstrated strong validity and reliability. The youth-report form of the MAPS and its short form are appropriate for use among children and adolescents experiencing acute clinical symptoms. The MAPS youth-report form will allow for nuanced, in-depth assessment of the parenting behaviors beyond parent-report that are critical to treatment outcomes in youth.

Parenting practices are primary targets of evidence-based psychosocial treatments for child and adolescent psychopathology, including conduct problems (Dishion et al., 2016; Forehand et al., 2013), autism (Bearss et al., 2018), attention-deficit/hyperactivity disorder (ADHD)(Haack et al., 2017; Pelham & Fabiano, 2008; Sibley et al., 2016), anxiety (Cobham et al., 2017; Lebowitz et al., 2014), obsessive-compulsive disorder (OCD) (Freeman et al., 2014), trauma (Gurwitch & Warner-Metzger, 2022), and depression (Luby et al., 2018; Wells & Albano, 2005). In addition to research identifying parenting as a critical mechanism of change in youth clinical outcomes, further evidence has demonstrated that parenting behaviors mediate the association between child symptoms and known risk factors such as parental symptomology. Parenting has been associated with children’s overall functioning, as well as disorders including ADHD, oppositional defiant disorder (ODD), anxiety, and depression, and across more broad symptoms of internalizing and externalizing (Breaux et al., 2017; Elgar et al., 2007; Goodman et al., 2020; Orgilés et al., 2018).

Despite ample evidence of parenting as a primary target in many youth psychosocial treatments and parenting practices as a clear mechanism of change in outcomes, formal assessment of parenting is often overlooked in clinical practice in favor of focusing on youth symptom outcomes (Hurley et al., 2014; O’Connell et al., 2015; O’Connor, 2002). The gold standard in parenting assessment, observational methods, is difficult to implement in standard care due to limitations on time and physical location. Further, live observation in a clinical care context may not fully capture a naturalistic range of parenting behaviors (e.g., yelling, physical punishment) due to clinician presence, either in-person or on telehealth platforms. Therefore, the most common method used when assessing parenting is parent-reported surveys. However, these surveys have historically demonstrated weak psychometric properties (e.g., low reliability, limited variability, or ceiling effects) (Hurley et al., 2014). Parent-report surveys also require multiple scales to assess a range of parenting skills that are positive (e.g., warmth, supportiveness) and/or negative (e.g., hostility, laxness), and do not easily capture parenting across developmental stages.

The Multidimensional Assessment of Parenting Scale (MAPS) was developed to overcome these limitations of parent-report parenting scales (Parent & Forehand, 2017). Using an empirically-based approach, the MAPS was created using items adapted from several well-established parenting scales. The MAPS factor structure includes a broadband positive parenting and a broadband negative parenting domain. Broadband positive parenting is comprised of four narrow-band scales: warmth, praise, proactive parenting, and supportiveness. Broadband negative parenting is comprised of three narrowband subscales: hostility, lax control, and physical control. The MAPS factor scores have shown strong psychometric properties, measurement invariance across three developmental stages (early childhood, middle childhood, and adolescence), and growing evidence for validity (Loiselle et al., 2021).

Although the MAPS successfully addressed important limitations of prior parent-reported parenting scales by examining a wide range of behaviors across positive and negative domains, over-reliance on parent reports alone remains a significant limitation. Youth perceptions of parenting are a critical aspect of parenting assessment, given that observed parenting behavior has been shown to converge more with youth reports than with parent reports (Parent et al., 2014). For example, the correlation between observed positive parenting and youth-reported positive parenting is twice as large as the correlation with parent-report. Assessment of negative parenting has shown to be more nuanced, with the more “accurate” informant (i.e., higher correlation with observed negative parenting) depending on individual differences in parents and youths, such as experiences of psychopathology symptoms (Parent et al., 2014). For example, youth who report higher depressive symptoms may overreport negative parenting relative to observed levels, whereas the opposite is true for parenting reports. Further, in some clinical settings (e.g., adolescent inpatient units) parental reports may be difficult to collect, whereas in other settings (e.g., family-based treatment) the discrepancy between parent and youth reports may be of primary clinical interest. Ultimately, parenting assessment is likely incomplete without assessing perceptions from both parent and youth reports; however, a comprehensive assessment of parenting practices that considers multiple informants has not been available. Thus, multi-informant assessment of parenting practices is a vital practice, necessary for monitoring progress and evaluating the outcomes of family-based treatments.

The current study aimed to expand the utility of the MAPS by adapting and evaluating it for youth-report, which provides an additional perspective on parenting practices. We evaluated the youth-report form of the MAPS in a large clinical population ranging from middle childhood (8–12) to adolescence (13–17). We hypothesized that the MAPS-Y (youth) form would demonstrate a similar factor structure as the parent form (warmth, praise, proactive parenting, and supportiveness grouped in positive parenting; hostility, lax control, and physical control grouped in negative parenting). Further, we hypothesized similarly strong reliability and initial support for validity. Next, we explored the MAPS-Y’s measurement invariance across developmental stages and its sensitivity to family-based intervention. Additionally, MAPS was administered to primary caregivers, and we examined the cross-informant agreement between parent and youth reports on parenting practices. Finally, to address the limitation of time constraints in a clinical setting and to develop a shorter form of the scale for progress monitoring, we adapted and tested a short form of the MAPS-Y and examined its similar to the full version of the MAPS-Y.

## Method

### Participants

A total of 628 youth who were admitted to intensive psychiatric units were included in the current study if they had completed the youth-report of the MAPS during their admission to an intensive program. Units included a child (7–12 years old) partial hospitalization program (CPHP) and an adolescent (11–18 years old) inpatient unit (AIU). The partial psychiatric hospitalization program is a 5-day-per-week intensive treatment program with hours typical of school settings, and children return home each night. The adolescent inpatient unit involves 24-hour psychiatric care in a structured hospital setting. Both treatment settings involve youth who are involuntarily admitted or whose families are seeking care to stabilize severe mental health crises and support recovery through therapy, medication, and multidisciplinary support. CPHP participants were admitted between September 2022 and September 2023, and AIU participants were admitted between October 2021 and June 2023.

Overall, youth across both samples (*M* age = 14, *SD* = 2.56) self-identified as cisgender female (41.0%), cisgender male (31.5%), or transgender, non-binary, or otherwise gender non-conforming (27.4%). Youth sex assigned at birth as reported on their hospital medical records was 36% male and 64% female. Further, youth race was self-reported as American Indian or Indigenous (6.1%), Asian (3.3%), Black (17.4%), White (63.1%), or as multiracial (10.1%) and youth ethnicity was self-reported as Latinx or Hispanic (29.5%) and non-Latinx or Hispanic (70.5%). As standard practice in the above clinical settings, parental report measures were also completed at admission by the self-identified primary caregiver. If two parents or caregivers completed the forms, the first to complete them was used in the current study.

### Ethics Statement

The Lifespan Institutional Review Board approved the study as a retrospective chart review (598992-39), and the measures were administered to inform clinical care in inpatient and partial hospitalization programs. This retrospective study of medical records was conducted in October 2023.

### Procedures

Participants completed the clinical assessment battery of measures within the first week of admission to one of the hospital programs. In the CPHP, parents also completed parallel measures of parenting and child functioning, which were not routinely collected in the inpatient setting at the time of data extraction. If the participants had more than one hospitalization during the study time frame, only the initial admission was evaluated. Specific measures included in the chart review from both hospitalization programs are detailed below. Beyond the MAPS, measures are included that have known associations with parenting practices, including internalizing and externalizing psychopathology, emotion regulation, sleep health, family context, and exposure to adversity.

### Measures

#### Multidimensional Assessment of Parenting Scale – Parent and Youth Report Forms

(MAPS) (Parent & Forehand, 2017). The MAPS assesses positive and negative parenting practices across seven domains (34 total items). The scale was developed from established measures of parenting practices to select optimal parenting items constituting both positive and negative dimensions of warmth/hostility and behavioral control appropriate for parents of children across the developmental span from young childhood through adolescence. The MAPS has demonstrated excellent internal and test-retest reliability as well as strong support for the validity of MAPS subscale scores (Parent & Forehand, 2017; Perry et al., 2023; Poppert Cordts et al., 2020). Additionally, measurement invariance across youth developmental stages from young childhood to adolescence has been established for the MAPS subscales. The Youth-report form of the MAPS is a direct adaptation of the MAPS to orient the questions to the youth perspective on the same parenting questions.

The Broadband Positive Parenting subscale of the MAPS includes four narrowband subscales: Proactive Parenting which measures child-centered appropriate responses to anticipated difficulties; Positive Reinforcement which measures contingent responses to positive child behavior with praise, rewards, or displays of approval; Warmth which measures displays of affection; and Supportiveness which measures displayed interest in the child, encouragement of positive communication, and openness to a child’s ideas and opinions.

The Broadband Negative Parenting factor includes three narrowband subscales: Hostility which includes items representing intrusive parenting that is over controlling and parent-centered as well as harshness which includes coercive processes such as arguing, threats, yelling, ineffective discipline, and irritability; Physical Control which includes items representing physical discipline both generally and specifically out of anger and frustration; and Lax Control which includes items representing permissiveness or the absence of control, easily coerced control in which the parent backs down from control attempts based on the child’s behavior, and inconsistency which is the failure to follow through with control or inconsistent applying consequences.

##### Adolescent (AIU) validity measures

**Psychopathology Symptoms**. The Suicidal Ideation Questionnaire- Junior (SIQ-JR) (Reynolds & Mazza, 1999) was used to measure youth-reported past month suicidal ideation. Likert scale responses capture the frequency of thoughts and range from 0 (*I’ve never had this thought*) to 6 (*Almost every day*). The Self-Injurious Thoughts and Behaviors Interview (SITBI) (Nock et al., 2007) was used to assess past month frequency of suicide attempts and non-suicidal self-injury. The Patient-Reported Outcomes Measurement Information System (PROMIS) short forms were used to assess youth-reported anxiety, depression, and anger symptom severity (Varni et al., 2014). Each short form ranges from 4 to 8 items in length and asks respondents to assess the presence of their symptoms within the past 7 days. Scores for anxiety, depressive symptoms, and anger are calculated using the same 5-point rating scale ranging from “Never” to “Almost Always”.

**Transdiagnostic Factors**. The 18-item short form of the Difficulties in Emotion Regulation Scale (DERS) (Gratz & Roemer, 2004) was used to assess youth-reported domains of emotion regulation on a 1 (Almost never) to 5 (Almost always) Likert Scale. Responses summed for a total score, with higher scores indicating greater difficulties with emotion regulation. The short-form PROMIS Stress and Sleep Disturbances scales were used to assess youth-reported subjective experiences of stress and difficulties with sleep quality or staying asleep. The PROMIS psychological stress (Bevans et al., 2018) and sleep disturbance (van Kooten et al., 2021) scales use 5-point rating scales that range from 1 (Never) to 5 (Always). Higher scores indicate higher levels of stress or sleep disturbances.

**Family Context and Adversity**. The 12-item version of the Family Assessment Device (FAD-12) (Boterhoven de Haan et al., 2015) was used to assess adolescent satisfaction with general family functioning. A 4-point Likert scale rated responses from “Strongly Disagree” to “Strongly Agree”. Sample items are “We cannot talk to each other about sadness we feel” (negative item), and “We are able to make decisions about how to solve problems” (positive item). Higher scores indicate more problematic overall family functioning. The Adverse Childhood Experiences Questionnaire (ACE-Q) (Purewal et al., 2016) was used to assess 19 adverse childhood experiences based on youth report. Adolescents indicated how many items they have experienced, and a total count of items experienced was used in the current study.

##### Child (CPHP) validity measures

**Psychopathology Symptoms**. The total score of the Screen for Child Anxiety Related Disorders (SCARED) (Birmaher et al., 1997) was used to assess youth-reported anxiety severity. A 3-point Likert scale ranged from “Not True or Hardly Ever True” to “Very True or Often True” with higher scores indicating more severe anxiety symptoms. The total score of the Children’s Depression Inventory 2nd Edition (CDI) (Kovacs, 2015) was used to assess youth-reported depressive symptom severity as well as a single item to assess suicidal ideation (“I do not think about killing myself”, “I think about killing myself but would not do it” or “I want to kill myself”). A 3-point item-specific Likert scale is used, with higher scores indicating more severe depressive symptoms. Parents completed the Disruptive Behavior Disorders (DBD) (Pelham et al., 1992) rating scale to assess parent-reported externalizing symptoms. For the current study, the oppositional defiant disorder subscale was used to assess oppositionality, and an irritability scale was also created based on common irritability symptoms used in other scales (e.g., “Is often angry or resentful”). A 4-point Likert scale ranging from “Not at all” to “Very much” is used with higher scores indicating more severe oppositional or irritable symptoms.

**Transdiagnostic Factors**. Youth completed the Children’s Emotion Management Scale (CEMS-Youth Report) (J. Zeman et al., 2001; J. L. Zeman et al., 2010). The emotional dysregulation subscales for worry, anger, and sadness were used to assess cross-cutting emotional dysregulation. Items are on a 3-point Likert-type scale ranging from “Hardly ever” to “Often” with higher scores indicating more severe emotion-specific dysregulation. Similar to the adolescent measures, the short-form of the PROMIS Sleep Disturbances scale was used to assess difficulties with youth-reported sleep quality or staying asleep (van Kooten et al., 2021).

**Family Context and Adversity**. The eight-item short-form of the PROMIS pediatric Family Relationships scale was used to assess youth perceptions of family connection and support. Similar to the FAD-12, the PROMIS scale measures youth perceptions of general family functioning. Items are rated on a 5-item Likert scale from “Never” to “Always” with higher scores reflecting higher quality family relationships. Parents completed the Center for Youth Wellness Adverse Childhood Experiences Questionnaire (CYW ACE-Q) (Harris & Renschler, 2015; Purewal et al., 2016) to assess youth exposure to adversity based on parental reports. A total of 17 ACEs were reported on, and a total score was used with higher scores indicating higher adversity.

### Data Analytic Plan

Analyses were conducted in four phases. In the first stage (model fit), confirmatory factor analysis was conducted based on the original parent-report factor structure of the MAPS. Analyses were conducted with the Lavaan R package using Jamovi statistical software. The following fit statistics were employed to evaluate model fit: chi-square, χ2: p > 0.05 excellent, comparative fit index (CFI; > 0.90 acceptable, > 0.95 excellent), root mean square error of approximation (RMSEA; < 0.08 acceptable, < 0.05 excellent), and the standardized root mean square residual (SRMR; < 0.08 acceptable, < 0.05 excellent). We also aimed for factor loadings above 0.40. Although participants were included in the current study if they completed the MAPS-Y, youth may have skipped some items on the scale, leading to missing data ranging from 0.0% to 2.1%. Missing data were handled using Full Information Maximum Likelihood (FIML), the default approach for maximum likelihood estimators, which uses all available data under the assumption that data are missing at random. Following confirmatory factor analysis (CFA) models, we examined three forms of measurement invariance using multiple group CFAs: configural, metric, and scalar. Measurement invariance was examined across youth race, ethnicity, and developmental stage (i.e., 7–12 and 13–18).

In stage two (reliability and agreement), Omega and alpha coefficients were estimated for internal consistency. Further, for the CPHP youth sample only, we examined the cross-informant agreement using bivariate correlations between youth and parent-report forms (*n* = 99). In stage three (validity), we examined correlations between youth MAPS factor scores and psychopathology, transdiagnostic factors, and family context and adversity domains separately by CPHP (7–12) and AIU (11–18) samples, given that the measures used differed among hospital programs. Finally, in stage four (short-form), we developed a short form of the MAPS youth report using a combination of item response theory (IRT), CFA, correlations, and Omega reliability to ensure maximum coverage, reliability, and validity of a short-form. The CPHP and AIU samples were merged for analyses of primary model fit and reliability but examined separately for validity analyses.

## Results

### Phase One: Model Fit

The seven-factor structure mirroring the parent-report form demonstrated good model fit, χ^2^ (474) = 1339, *p* < *0*.001, RMSEA = 0.054, 90% CI [0.051, 0.057], CFI = 0.92, SRMR = 0.059, see Table 1 for complete CFA results. Furthermore, all items had a significant factor loading above 0.40 except for item 23 on the Lax Control subscale. Regarding measurement invariance, scale invariance was met for race and ethnicity (all χ^2^ difference test *p*s > 0.05), but not for the developmental stage. For the developmental stage, only metric invariance was met (χ^2^ difference tests between metric and scale *p* < 0.05). However, ΔCFI was < 0.010, ΔRMSEA < 0.015, and ΔSRMR < 0.010, suggesting some support for strong invariance. To identify sources of bias across developmental stages, we examined modification indices (MIs) for item intercepts. Two items, MAPS07 (“My parent has warm and intimate times together with me”) and MAPS29 (“When my parent is upset or under stress, my parent is picky and on my back”), showed a meaningful MI (>10), indicating a violation of scalar invariance for the intercepts of those two items. Once those two item intercepts were freely estimated across groups, the partial scalar invariance did not fit significantly worse than the metric model (χ²(25) = 35.71, p = 0.076), indicating that scalar invariance held across developmental stages for the majority of items.

**Table 1:** CFA item loadings for MAPS youth-form subscales

Factor	Indicator	Estimate	SE	95% CI		Z	p	Stand. Estimate
				Lower	Upper			
PP	maps15	0.84	0.04	0.75	0.94	17.00	< 0.001	0.65
	maps19	0.87	0.05	0.77	0.97	17.13	< 0.001	0.65
	maps28	0.85	0.04	0.75	0.95	17.07	< 0.001	0.65
	maps32	0.91	0.04	0.82	1.00	19.92	< 0.001	0.73
	maps33	0.80	0.04	0.70	0.89	16.83	<0.001	0.65
PR	maps11	0.93	0.04	0.83	1.02	19.56	<0.001	0.71
	maps18	1.01	0.04	0.92	1.09	23.70	<0.001	0.81
	maps26	1.09	0.04	0.99	1.19	22.29	<0.001	0.78
	maps30	0.99	0.04	0.90	1.09	20.42	<0.001	0.73
WM	maps01	1.07	0.04	0.98	1.16	23.55	<0.001	0.81
	maps07	0.96	0.04	0.87	1.06	20.19	<0.001	0.73
	maps21	1.19	0.04	1.10	1.28	26.93	<0.001	0.89
SP	maps10	1.07	0.04	0.98	1.16	23.25	<0.001	0.80
	maps17	0.92	0.04	0.83	1.01	19.44	<0.001	0.70
	maps22	1.06	0.04	0.98	1.14	25.26	<0.001	0.84
HS	maps04	0.88	0.04	0.79	0.96	20.69	<0.001	0.73
	maps05	0.97	0.04	0.87	1.06	19.98	<0.001	0.71
	maps06	0.98	0.05	0.88	1.08	19.29	<0.001	0.69
	maps08	1.01	0.04	0.92	1.10	21.89	<0.001	0.76
	maps13	1.16	0.04	1.08	1.25	26.94	<0.001	0.87
	maps16	1.15	0.04	1.06	1.23	25.39	<0.001	0.84
	maps29	1.03	0.05	0.93	1.14	19.93	<0.001	0.71
LC	maps02	0.69	0.04	0.60	0.79	13.98	<0.001	0.56
	maps03	0.59	0.04	0.49	0.68	12.56	<0.001	0.51
	maps09	0.76	0.04	0.66	0.85	16.15	<0.001	0.64
	maps12	0.69	0.04	0.60	0.79	14.33	<0.001	0.58
	maps20	0.76	0.03	0.68	0.83	19.75	<0.001	0.74
	maps23	0.44	0.04	0.34	0.54	8.93	<0.001	0.38
	maps27	0.71	0.03	0.63	0.79	18.33	<0.001	0.70
PC	maps14	0.82	0.03	0.76	0.88	26.07	<0.001	0.85
	maps24	0.89	0.03	0.83	0.96	26.57	<0.001	0.86
	maps25	0.95	0.03	0.88	1.01	27.79	<0.001	0.89
	maps31	0.88	0.03	0.81	0.94	27.45	<0.001	0.88

*CI* confidence interval, *HS* hostility, *LC* lax control, *PC* physical control, *PP* proactive parenting, *PR* positive reinforcement, *SE* standard error, *SP* supportiveness, *WM* warmth

### Phase Two: Reliability and Agreement

Reliability was strong for all scales (see Table 2 for reliability, descriptive statistics, and correlations between subscales). Specifically, reliability was excellent for proactive parenting (Ω = 0.828), positive reinforcement (Ω = 0.847), warmth (Ω = 0.853), supportiveness (Ω = 0.831), hostility (Ω = 0.908), and physical control (Ω = 0.928) and was acceptable for lax control (Ω = 0.797). Reliability for the broadband positive (Ω = 0.925) and negative (Ω = 0.880) scales was also excellent. Regarding the cross-informant agreement in the child CPHP subsample where both youth and parent report version of the MAPS were administered, the correlation between youth and parent reports was modest for positive parenting (*r* = 0.220, *p* < 0.05) and negative parenting (*r* = 0.220, *p* < 0.05), which is consistent with past parent-youth agreement research on parenting and mental health (De Los Reyes et al., 2015).

**Table 2:** Mean, standard deviations, reliability, and correlations between youth-report narrowband and broadband scales

	M (SD)	Omega	PP	PR	WM	SP	HS	LC	PC	POS
PP	2.94 (0.94)	0.82	—							
PR	3.07 (1.10)	0.84	0.64	—						
WM	3.10 (1.17)	0.85	0.45	0.59	—					
SP	3.22 (1.12)	0.83	0.62	0.72	0.64	—				
HS	2.82 (1.09)	0.90	−0.09	−0.22	−0.24	−0.37	—			
LC	2.04 (0.75)	0.80	0.24	0.26	0.17	0.15	0.21	—		
PC	1.52 (0.92)	0.92	−0.06	−0.14	−0.11	−0.21	0.46	0.17	—	
POS	3.06 (0.89)	0.92	0.78	0.87	0.81	0.89	−0.28	0.25	−0.16	—
NEG	2.23 (0.68)	0.88	0.01	−0.08	−0.11	−0.24	0.82	0.56	0.76	−0.12

*HS* hostility, *LC* lax control, *M* mean, *NEG* broadband negative parenting, *PC* physical control, *POS* broadband positive parenting, *PP* proactive parenting, *PR* positive reinforcement, *SD* standard deviation, *SP* supportiveness, *WM* warmth

### Phase Three: Validity

Table 3a and b include correlations across the adolescent and child samples, respectively. In adolescence, higher levels of proactive parenting were associated with higher perceived stress, lower adversity, and lower family relationship quality. Unexpectedly, higher levels of proactive parenting were related to higher levels of past suicide attempts, though this could be consistent with a need for more proactive parenting practices when youth are at risk for self-harm. Higher levels of positive reinforcement, warmth, and supportiveness were related to higher adolescent self-compassion and perceived family relationship quality as well as lower levels of adversity. Further, higher levels of supportiveness were related to lower levels of adolescent emotion dysregulation. Regarding negative parenting, higher levels of hostility were associated with higher levels of depressive symptoms, suicidal ideation, suicide attempts, anxiety, irritability, emotion dysregulation, sleep disturbances, adversity, and stress. Further, higher hostility was also related to lower adolescent self-compassion and lower quality family relationships. More lax control was associated with higher levels of depressive symptoms, anxiety, irritability, and emotion dysregulation but was the only subscale not associated with family relationship quality or adversity. Finally, more physical control was associated with higher depressive symptoms, anxiety, irritability, emotion dysregulation, adversity, and sleep disturbance as well as lower levels of family relationship quality.

**Table 3:** **a** Adolescent inpatient unit validation: ages 13–18 years; **b** Child partial hospital program validation: ages 7–12 years

	PP	PR	WM	SP	HS	HSs	LC	LCs	PC	POS	POSs	NEG
a - Adolescent Sample												
Depression	0.07	−0.03	−0.01	−0.08	0.34*	0.30*	0.10*	0.08	0.11*	−0.02	−0.04	0.27*
SITBI	0.16*	0.09	0.09	0.10	0.17*	0.15*	0.04	−0.01	−0.01	0.13*	0.11	0.11*
SIQ	0.05	−0.06	−0.02	−0.07	0.30*	0.24*	0.06	0.02	0.06	−0.03	−0.05	0.22*
Anxiety	0.08	−0.01	0.01	−0.07	0.32*	0.28*	0.10*	0.09*	0.16*	−0.01	−0.02	0.28*
Irritability	0.06	0.01	−0.04	−0.08	0.34*	0.32*	0.18*	0.16*	0.15*	−0.02	−0.02	0.32*
DERS	0.04	−0.06	−0.06	−0.14*	0.37*	0.32*	0.15*	0.12*	0.13*	−0.07	−0.08	0.31*
Self-com	0.08	0.16*	0.10*	0.15*	−0.19*	−0.14*	0.04	0.06	−0.09*	0.15*	0.14*	−0.13*
Sleep	0.01	−0.04	−0.05	−0.10*	0.30*	0.29*	0.04	0.01	0.13*	−0.06	−0.06	0.24*
Stress	0.09*	−0.01	0.02	−0.02	0.34*	0.29*	0.10*	0.07	0.05	0.02	−0.01	0.24*
ACE	−0.12*	−0.14*	−0.22*	−0.23*	0.32*	0.29*	−0.01	−0.02	0.19*	−0.21*	−0.22*	0.25*
FAD	−0.38*	−0.51*	−0.54*	−0.64*	0.59*	0.56*	−0.05	−0.07	0.33*	−0.61*	−0.64*	0.44*

b - Child Sample												
Depression	−0.21*	−0.35*	−0.35*	−0.30*	0.39*	0.32*	0.01	−0.03	0.19*	−0.38*	−0.38*	0.28*
SI	−0.23*	−0.29*	−0.29*	−0.28*	0.42*	0.35*	0.01	−0.04	0.28*	−0.34*	−0.33*	0.34*
Anxiety	−**0.05**	−0.14*	−0.28*	−0.18*	0.40*	0.34*	0.21*	0.12	0.25*	−0.21*	−0.18*	0.39*
Irritability	0.01	−0.06	−0.11	−0.11	0.20*	0.18*	0.15	0.11	0.09	−0.09	−0.05	0.19*
Oppositional	0.03	−0.08	−0.13	−0.13	0.25*	0.24*	0.21*	0.19	0.10	−0.10	−0.05	0.25*
Worry Dysreg	0.08	0.07	0.01	0.05	0.13	0.11	0.08	0.09	0.03	0.06	0.05	0.11
Sad Dysreg	0.17*	0.01	0.16	0.04	0.14	0.09	0.12	0.08	0.07	0.11	0.09	0.14
Anger Dysreg	−0.02	−0.03	−0.08	−0.07	0.31*	0.30*	0.05	0.06	0.26*	−0.07	−0.08	0.30*
Sleep	−0.11	−0.10	−0.19*	−0.11	0.24*	0.21*	0.09	0.05	0.01	−0.16*	−0.15*	0.16*
ACE	0.03	−0.00	−0.02	0.11	−0.11	−0.14	0.03	−0.02	−0.09	0.03	0.01	−0.07
Family	0.30*	0.55*	0.51*	0.57*	−0.44*	−0.38*	0.07	0.10	−0.19*	0.62*	0.58*	−0.28*

*ACE* adverse childhood experiences, *DERS* difficulties in emotion regulation scale, *FAD* family assessment device, *HS* hostility, *LC* lax control, *NEG* broadband negative parenting, *PC* physical control, *POS* broadband positive parenting, *POSs* POS short form, *PP* proactive parenting, *PR* positive reinforcement, *SITBI* self-injurious thoughts and behaviors interview, *Self-com* self-compassion, *SP* supportiveness, *WM* warmth, *SI* suicidal ideation

*p < 0.05

In the childhood partial sample (ages 8–12), higher levels of proactive parenting, positive reinforcement, warmth, and supportiveness were related to lower depressive symptoms, suicidal ideation, and anxiety as well as higher family relationship quality. In addition, higher levels of warmth were related to lower levels of sleep disturbances, and higher levels of proactive parenting were related to high sadness dysregulation. Similar to adolescent results, higher levels of hostility were related to higher child depressive symptoms, suicidal ideation, anxiety, irritability, and sleep disturbances as well as lower family relationship quality. Further, higher hostility was associated with higher oppositionality and anger dysregulation. Higher lax control was only associated with higher anxiety and oppositionality. Lastly, more physical control was related to higher depressive symptoms, suicidal ideation, anxiety, and anger dysregulation as well as lower family relationship quality.

### Phase Four: Short-Form Development of the MAPS-Y

We began development of the short-form of the youth report version of the MAPS by using an IRT graded response model for each narrowband subscale. We then selected items with the highest discrimination parameters for inclusion in the short-form. For positive parenting, we sought to create a single broadband scale given that narrowband scales were already short. Based on discrimination parameters, we selected six items for the broadband positive parenting short-form that covered each of the narrowband domains. Regarding negative parenting, we developed a short-form of the hostility and lax control subscales, each with three items with the highest IRT discrimination parameters. A total of 12 items were selected for the short-form. We then conducted a CFA with all three short-form scales and model fit was good, χ^2^ (51) = 224, *p* < *0*.001, RMSEA = 0.074, 90% CI [0.064, 0.083], CFI = 0.948, SRMR = 0.051. Across scales, standardized factor loadings ranged from 0.558 to 0.888 (see Table 4). Reliability for the youth-report short form was excellent for short-form positive parenting (Ω = 0.871) and hostility (Ω = 0.874) and was acceptable for lax control (Ω = 0.754). Correlations between full and short-form scales were *r* = 0.940 for positive parenting, *r* = 0.934 for hostility, and *r* = 0.894 for lax control. Parent-youth agreement for the short-form was similar to the full version and ranged from *r* = 0.256 to *r* = 0.317. Finally, patterns of correlations with youth and family outcomes were similar to the full version and are presented in 3a and 3b.

**Table 4:** Short-form confirmatory factor analysis results

Short-form Factor Loadings								
Factor	Indicator	Estimate	SE	95% Confidence Interval		Z	p	Stand. Estimate
				Lower	Upper			
PosPar	maps22	1.02	0.04	0.94	1.11	23.7	<0.001	0.81
	maps32	0.739	0.04	0.64	0.83	15.4	<0.001	0.59
	maps18	0.93	0.04	0.84	1.02	20.8	<0.001	0.75
	maps21	0.93	0.04	0.83	1.02	19.0	<0.001	0.69
	maps10	1.04	0.04	0.95	1.13	22.1	<0.001	0.77
	maps26	1.02	0.05	0.92	1.12	20.0	<0.001	0.73
HS	maps08	1.00	0.04	0.91	1.09	21.2	<0.001	0.75
	maps13	1.18	0.04	1.10	1.27	26.3	<0.001	0.88
	maps16	1.16	0.04	1.07	1.26	24.9	<0.001	0.85
LC	maps09	0.66	0.05	0.56	0.76	13.2	<0.001	0.55
	maps20	0.78	0.04	0.70	0.87	18.5	<0.001	0.77
	maps27	0.78	0.04	0.70	0.86	18.6	<0.001	0.77

*HS* hostility, *LC* lax control, *PosPar* positive parenting, *SE* standard error

## Discussion

The current study evaluated the youth-report version of the MAPS with the overall goal of identifying a reliable and valid measure of parenting behavior as reported by a clinical sample of youths. Confirmatory factor analyses and model fit indices demonstrated that all items included in the survey loaded onto subscales as expected and with good fit, providing support for use of the same factor structure in the MAPS youth-report as in the parent-report version. As hypothesized, analyses demonstrated strong reliability and validity of the measure, establishing the youth-report version as a useful measure of parenting behaviors in a clinical sample.

An integral objective for the MAPS youth report was addressing the field’s current dearth of multi-informant measures of parenting behaviors. Analyses of convergence across reporters demonstrated modest agreement between parent and youth reports (ages 8–12) for positive and negative parenting behaviors. These findings provide further confidence in the measure’s ability to address the scarcity of parenting assessments that collect the perspectives of both youth and parents. These results also align with existing research and theory for youth and parent agreement on parenting measures (Korelitz & Garber, 2016; Parent et al., 2014). Future research should examine the clinical implications of informant discrepancies on parenting practices. When agreement among informants is high, it may indicate attunement, whereas significant discrepancies could suggest high parent-child conflict or perceptual biases of one or both informants. Additionally, discrepancies may serve as a potential target in family-based treatments to increase alignment, enhance communication, and develop shared family values or goals. For example, when multiple informants are collected as part of routine care, scoring software could provide the degree to which parents and youth agree, which could point to possible areas for further assessment (e.g., interviews, observational tasks) or intervention.

In the current study, we examined the youth-report scale across multiple developmental stages, similar to the original parent-reported version. Importantly, the factor structure did not vary based on the respondents’ age (i.e., child or adolescent sample), and full scalar invariance was achieved across racial and ethnic groups, indicating a significant strength of this measure as applicable across developmental stages and diverse racial backgrounds. However, only metric invariance was achieved when comparing middle childhood (7–12) and adolescence (13–17) reports, even though the change in model fit (i.e., ΔCFI < 0.010, ΔRMSEA < 0.015, and ΔSRMR < 0.010) was within recommended thresholds that support partial or approximate scalar invariance. Researchers can confidently compare associations between constructs across age groups (e.g., correlations, regressions) because the factor loadings are equivalent. However, we recommend that clinicians and researchers consider using age-based normed T-scores (provided in the supplemental appendix) when comparing youth reports of parenting practices across childhood and adolescence, as the same raw score might reflect different underlying trait levels depending on age. Additionally, although we found evidence of measurement invariance across racial and ethnic groups in the current sample, it remains essential for future research to examine the psychometric properties of the MAPS-Y in broader and more diverse contexts—such as among youth from different cultural backgrounds, in non-clinical settings, and across various languages—to ensure reliable and valid interpretation of scale scores across populations.

Validity of the measure was tested by examining associations between MAPS youth-report subscales and youth-reported internalizing and externalizing symptoms. Contrary to hypotheses, not all subscales demonstrated strong associations with the expected symptoms across the child and adolescent samples. For example, in the adolescent sample, high hostility was related to high self-compassion and higher quality family relationships. In the child sample, high proactive parenting was related to high sadness dysregulation. Further, several associations were found between internalizing and externalizing symptoms in both adolescent and child samples. Among adolescents, high hostility on the negative parenting scale was related to higher anxiety and depression. Similarly, for the child sample, proactive parenting was associated with lower depression and anxiety. Additionally, high hostility was related to higher depression and anxiety. These correlations suggest that some symptoms may be more predictive of certain subscales. However, consistent associations were found between symptoms and both the hostility and broadband negative parenting scales, suggesting that parenting-based interventions may specifically target hostility within negative parenting. Finally, examination of a short form of the MAPS-Y demonstrated that the short form reliably measures each of the three parenting domains, shows agreement with parents and youths, and correlates with clinical measures comparably to the full version. These findings strengthen the MAPS-Y’s applicability and utility in clinical contexts, where time is often a constraint for assessments.

Limitations of the current study should also be noted. The use of a clinical, hospital-based sample allows for the much-needed use of the MAPS youth-report across acute and clinical populations, but the use of the measure in non-clinical samples is therefore not generalizable from these results. Future research should examine the MAPS youth-report in community samples to allow for the widespread use of a multi-informant parent behavior measurement across care settings. Additionally, this study combined responses from participants across childhood and adolescence. While this approach allows for broad application of the measure across developmental stages, future research may explore the factor structure individually in childhood and adolescence to determine the need for higher specificity of reporters (i.e., child version and adolescent version). Another limitation of the current study is the absence of observational methods to compare youth self-reports, as well as the lack of data regarding treatment outcomes or progress monitoring to assess the sensitivity of scores to changes in intervention. Future research should compare youth and parent report forms with observational methods and evaluate how the MAPS scale scores change in response to family-based intervention, treating this as a treatment outcome and utilizing the short-form for weekly progress tracking.

Taking these findings together, the current study demonstrates the youth-report and youth-report short form of the MAPS as appropriate for use among children and adolescents experiencing acute clinical symptoms. The factor structure of the youth-report was invariant across developmental stages, included both positive and negative domains, and evidenced strong psychometric properties. The development of the youth-report is a much-needed addition to the parent-reported MAPS, and its use will allow for nuanced, in-depth assessment of the parenting behaviors that are so critical to youth outcomes.

## Supplementary information

Supplementary materials
