# Supplementary material for: The Multidimensional Assessment of Parenting Scale: Youth Report Form in Inpatient and Partial Hospital Settings
Source: J Child Fam Stud. 2025 Aug 12;34(8):2209–19. doi: 10.1007/s10826-025-03131-x (PMC12394287; doi:10.1007/s10826-025-03131-x)
Supplement: Supplementary file 1 — Supplementary materials [file 10826_2025_3131_MOESM1_ESM.docx]

| **Instructions:**  Parents have different ways of trying to raise their children. Please read each statement and rate how much each one best describes your parent during the **past two months**. |  | | | | |
| --- | --- | --- | --- | --- | --- |
|  | **Never** | **Almost Never** | **Sometimes** | **Often** | **Always** |
| 1. My parent shows affection by hugging, kissing, and holding me. | 1 | 2 | 3 | 4 | 5 |
| 1. If I whine or complain when my parent takes away a privilege, he/she will give it back. | 1 | 2 | 3 | 4 | 5 |
| 1. My parent is afraid that disciplining me for misbehavior will cause me to not like him/her. | 1 | 2 | 3 | 4 | 5 |
| 1. My parent argues with me. | 1 | 2 | 3 | 4 | 5 |
| 1. My parent uses threats as punishment with little or no justification. | 1 | 2 | 3 | 4 | 5 |
| 1. The punishment my parent gives me depends on my parent’s mood. | 1 | 2 | 3 | 4 | 5 |
| 1. My parent has warm and intimate times together with me. | 1 | 2 | 3 | 4 | 5 |
| 1. My parent yells or shouts when I misbehave. | 1 | 2 | 3 | 4 | 5 |
| 1. I talk my parent out of punishing me after I have done something wrong. | 1 | 2 | 3 | 4 | 5 |
| 1. My parent shows respect for my opinions by encouraging me to express them. | 1 | 2 | 3 | 4 | 5 |
| 1. If I do my chores, my parent will recognize my behavior in some manner. | 1 | 2 | 3 | 4 | 5 |
| 1. My parent lets me out of a punishment early (like lift restrictions earlier than my parent originally said). | 1 | 2 | 3 | 4 | 5 |
| 1. My parent explodes in anger towards me. | 1 | 2 | 3 | 4 | 5 |
| 1. My parent spanks me with his/her hand when I have done something wrong. | 1 | 2 | 3 | 4 | 5 |
| 1. My parent gives reasons for his/her requests (such as "We must leave in five minutes, so it's time to clean up."). | 1 | 2 | 3 | 4 | 5 |
| 1. My parent loses his/her temper when I don’t do something he/she asked me to do. | 1 | 2 | 3 | 4 | 5 |
|  | **Never** | **Almost Never** | **Sometimes** | **Often** | **Always** |
| 1. My parent encourages me to talk about my troubles. | 1 | 2 | 3 | 4 | 5 |
| 1. If my parent gives me a request and I carry out the request, my parent praises me for listening and complying. | 1 | 2 | 3 | 4 | 5 |
| 1. My parent warns me before a change of activity is required (such as a five-minute warning before leaving the house in the morning). | 1 | 2 | 3 | 4 | 5 |
| 1. If I get upset when my parent says “No,” my parent backs down and gives in to me. | 1 | 2 | 3 | 4 | 5 |
| 1. My parent and I hug and/or kiss each other. | 1 | 2 | 3 | 4 | 5 |
| 1. My parent listens to my ideas and opinions. | 1 | 2 | 3 | 4 | 5 |
| 1. My parent feels that getting me to obey is more trouble than it’s worth. | 1 | 2 | 3 | 4 | 5 |
| 1. My parent spanks me when he/she is extremely angry. | 1 | 2 | 3 | 4 | 5 |
| 1. My parent uses physical punishment as a way of disciplining me. | 1 | 2 | 3 | 4 | 5 |
| 1. If I clean my room, my parent will tell me how proud they are. | 1 | 2 | 3 | 4 | 5 |
| 1. My parent gives in to me when I cause a commotion about something. | 1 | 2 | 3 | 4 | 5 |
| 1. My parent tells me his/her expectations regarding behavior before I engage in an activity. | 1 | 2 | 3 | 4 | 5 |
| 1. When my parent is upset or under stress, my parent is picky and on my back. | 1 | 2 | 3 | 4 | 5 |
| 1. My parent tells me that he/she likes it when I help out around the house. | 1 | 2 | 3 | 4 | 5 |
| 1. My parent uses physical punishment (for example, spanking) to discipline me because other things my parent has tried have not worked. | 1 | 2 | 3 | 4 | 5 |
| 1. My parent provides me with a brief explanation when he/she disciplines my misbehavior. | 1 | 2 | 3 | 4 | 5 |
| 1. My parent avoids struggles with me by giving clear choices. | 1 | 2 | 3 | 4 | 5 |
| 1. When I misbehave, my parent lets me know what will happen if I don't behave. | 1 | 2 | 3 | 4 | 5 |
